# Effects of Alcell Lignin Methylolation and Lignin Adding Stage on Lignin-Based Phenolic Adhesives

**DOI:** 10.3390/molecules26226762

**Published:** 2021-11-09

**Authors:** Xianbin Ai, Shanghuan Feng, Tao Shui, Himant Kakkar, Chunbao Charles Xu

**Affiliations:** 1 Institute of Energy Research, Jiangxi Academy of Science, Nanchang 330096, China; aixb@jxas.ac.cn; 2 Department of Chemical and Biochemical Engineering, Western University, London, ON N6A 5B9, Canada; tshui@ualberta.ca (T.S.); himant.92@hotmail.com (H.K.)

**Keywords:** Alcell lignin, lignin methylolation, phenolic adhesives, lignin based phenolic adhesives

## Abstract

To investigate the effects of lignin methylolation and lignin adding stage on the resulted lignin-based phenolic adhesives, Alcell lignin activated with NaOH (AL) or methylolation (ML) was integrated into the phenolic adhesives system by replacing phenol at various adhesive synthesis stages or directly co-polymerizing with phenolic adhesives. Lignin integration into phenolic adhesives greatly increased the viscosity of the resultant adhesives, regardless of lignin methylolation or adding stage. ML introduction at the second stage of adhesive synthesis led to much bigger viscosity than ML or AL introduction into phenolic adhesives at any other stages. Lignin methylolation and lignin adding stage did not affect the thermal stability of lignin based phenolic adhesives, even though lignin-based adhesives were less thermally stable than NPF. Typical three-stage degradation characteristics were also observed on all the lignin-based phenolic adhesives. Three-ply plywoods can be successfully laminated with lignin based adhesives, and it was interesting that after 3 h of cooking in boiling water, the plywoods specimens bonded with lignin-based phenolic adhesives displayed higher bonding strength than the corresponding dry strength obtained after direct conditioning at 20 °C and 65% RH. Compared with NPF, lignin introduction significantly reduced the bonding strength of lignin based phenolic adhesives when applied for plywood lamination. However, no significant variation of bonding strength was detected among the lignin based phenolic adhesives, regardless of lignin methylolation or adding stages.

## 1. Introduction

Lignin has been considered as an alternative and renewable source for bio-materials and biochemicals in the past decades. As the second most abundant biopolymer from pulping and bioethanol industries, lignin displays an extremely complex structure depending on the species of plant sources and isolation process. Organosolv processes using organic solvents have been widely applied for lignocellulosic biomass fractionation [1,2]. As a typical technical lignin, organosolv lignin displays a variety of advantages such as high purity, low molecular weight and relatively narrow molecular weight distribution [3,4]. In contrast to cellulose, lignin is currently mainly applied for energy recovery in mills and thus is a vastly underutilized as a polymer.

The phenolic structure of lignin has been interesting to both academic and industrial communities as this phenolic structure makes lignin particularly suitable for preparing high strength polymeric materials, especially thermosetting resins such as phenolic resins, epoxy resins, etc. [5]. Lignin-based PF resins in which phenol is partially replaced with lignin have been extensively and intensely investigated by both academia and industry. While according to the study of Marko et al., even a small amount of lignin (3.4–9.4%) addition could promote the condensation reaction for PF resole synthesis, which was demonstrated by the increased molar masses and relatively high ratios of methylene bridges to the sum of free ortho- and para-aromatic groups [6]. Moreover, lignin is highly reactive to phenolic resin prepolymers. When phenolic adhesive system containing 20% lignin and 80% phenolic resin is used for automotive brake pads, competitive advantages are demonstrated when compared with the brake pads prepared with pure phenolic resin [7].

Although lignin reacts with formaldehyde, its reactivity towards formaldehyde is relatively low due to the limited reactive sites and the steric impediments of side chains in lignin structure. Therefore, lignin modifications such as demethylation [8], phenolation [9,10] and methylolation are essential to improve lignin applicability for PF resins. By forming catechol moieties in lignin macromolecule, thus reducing the steric hinderance of methyoxyl groups, demethylation of lignin could effectively increase the reactivity of lignin. Demethylation can sharply decrease the relative amount of the methoxy groups and increase the amount of phenolic hydroxyl groups. Demethylated lignin based PF adhesives with 60% phenol replacement contains less free formaldehyde or free phenol, but higher bonding strength compared with the PF adhesive prepared with unmodified lignin [8]. Phenolation effectively improves lignin reactivity by introducing more reactive functional groups. Phenolated lignin-based phenolic resins demonstrated superior quality to unmodified lignin based phenolic adhesives in both wet and dry bonding strengths [11]. Different from lignin phenolation, methylolation enhances lignin reactivity by introducing methylol groups into one or two aromatic carbon of the basic lignin structures [12,13,14]. Methylolated lignin is generated in basic condition. During the methylolation, one or two formaldehyde molecules is added on to the orth-C by replacing orth-H, forming methylol groups that can further react with free phenol [15]. An increase of the reaction order to 100% was observed in methylolated lignin-based phenolic resin curing reaction [16].

In our earlier study, organosolv lignin fractionated from cornstalk was activated and condensed directly with phenol formaldehyde adhesive for laminating plywood. When condensing with neat phenol formaldehyde adhesive, the formed glueline in laminated plywoods even demonstrated better bonding strength than that formed with neat phenol formaldehyde adhesive [17]. In this study, lignin activated by sodium hydroxide and methylolation are integrated into phenolic adhesives at various stages, to investigate the effects of lignin methylolation and lignin adding stage during adhesives synthesis on the resulted lignin based phenolic adhesives with respects to thermal stability and bonding strength.

## 2. Materials and Experiments

### 2.1. Materials

Phenol, formaldehyde solution (~37.0 wt%) and sodium hydroxide solution (50 wt%) were purchased from was purchased from Sigma-Aldrich, (Oakville, ON, Canada). Alcell lignin was provided by Lignol and its chemical composition is shown in Table 1. All the materials were used as received without further treatments.

### 2.2. Experiments

#### 2.2.1. Preparation of Neat Phenol Formaldehyde Adhesive

Neat phenol formaldehyde (NPF) adhesive was synthesized at an F/P molar ratio of 1.8 with the catalysis of sodium hydroxide. During the synthesis, 200 g of phenol, 85.1 g of water and 60 g of 50% sodium hydroxide solution were loaded into a 1000 mL three-neck glass reactor and heated to 60 °C. During the heating, 310.5 g of 37% formaldehyde solution was slowly added into the glass reactor. The reactor was then heated at 60 °C for 60 min, then heated at 80 °C for 180 min. The temperature was finally increased to 90 °C and maintained at 90 °C for 30 min, then the reactor was cooled down to room temperature to terminate the synthesis reaction.

#### 2.2.2. Preparation of Lignin-Based Phenolic Adhesives

**Figure 1:**
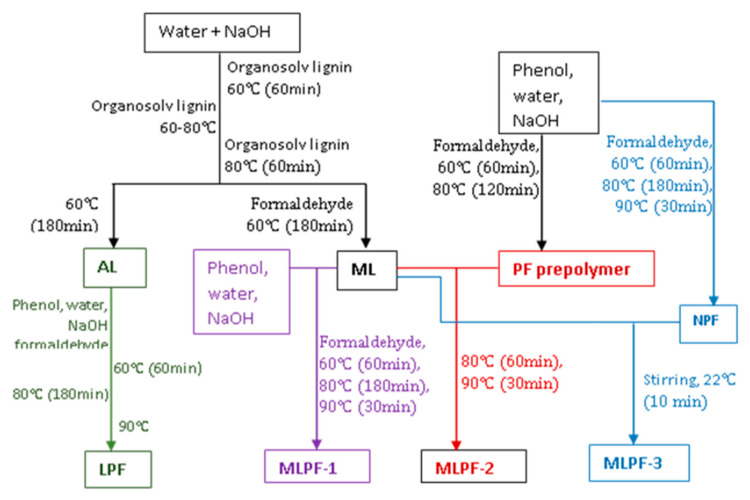
Diagram for phenolic adhesives preparation.

As Figure 1 illustrates, four types of Alcell lignin-based phenolic adhesives were prepared. Before the preparation of the lignin-based adhesives, aqueous lignin solution was first prepared: 200 g of Alcell lignin, 200 g of distilled water and 100 g of 50% NaOH solution was cooked, where lignin was added at three stages. Briefly, 100 g of 50% NaOH solution was mixed with 200 g of distilled water in a 1000 mL three-neck glass reactor under agitation. After 10 min of stirring, 80 g of Alcell lignin was added into the reactor. The reactor was then heated to 60 °C and held for 60 min. Sixty grams of the second portion of lignin was loaded into the reactor and the reactor was heated at 80 °C for 10 min, then 60 g of the third portion of the lignin was added. Finally, the whole mixture was heated at 80 °C for 60 min and then cooled down to room temperature. The obtained lignin solution was designated as AL. While for the preparation of methyloated lignin (ML) solution, AL was first heated to 60 °C, followed by a dropwise addition of 54.05 g of 37% formaldehyde solution. The mixture was then heated at 60 °C for 180 min.

As for AL-based adhesive synthesis, 250 g of AL, 125 g of phenol, 53.25 g of water and 6.25 g of 50% NaOH was first loaded into a 1000 mL three-neck glass reactor. The reactor was then heated at 60 °C. After 60 min, 227.9 g of 37% formaldehyde solution was loaded and further held at 60 °C for another 60 min. The heating temperature was then elevated to 80 °C and maintained at 80 °C. After 180 min, temperature was further increased to 90 °C and kept for 30 min. The resulted black viscous product was designated as LPF. In addition to LPF, three ML-based adhesives were also prepared. For MLPF-1 preparation, 153.95 g of ML was loaded into a 1000 mL three neck glass reactor connected to a condenser and a thermometer, followed by adding 50 g of phenol, 21.3 g of water and 5.0 g of 50% sodium hydroxide. The mixture was then heated to 60 °C. During the heating process, 77.65 g of 37% formaldehyde solution was loaded via a dropping funnel and the mixture was heated at 60 °C for 60 min, then heated at 80 °C for 180 min. The mixture was further heated at 90 °C for 30 min and the reactor was cooled down to room temperature to end the preparation process. As for MLPF-2 synthesis, 100 g of phenol, 42.6 g of water and 10 g of 50% sodium hydroxide were loaded into a 1000 mL three-neck glass reactor. The reactor was then heated to 60 °C. During the heating process, 155.3 g of 37% formaldehyde solution was loaded and heated at 60 °C for 60 min. The mixture was then heated up to 80 °C and maintained at 80 °C for 120 min. ML (307.9 g) was then loaded into the reactor. After heating at 80 °C for 60 min, the temperature was further elevated to 90 °C and heated for 30 min. MLPF-3 was prepared via a simple blending of ML and NPF at the weight ratio of 1:1 under agitation for 10 min at room temperature.

#### 2.2.3. Characterizations of the Adhesives

##### Basic Characterizations

Viscosity of the adhesives was tested with a brook viscometer (CAP 2000+ Viscometer, Brookfield, Middleboro, MA, USA) equipped with cone spindle at the 500 rpm of rotation speed at 50 °C Non-volatile content of the adhesives was determined by heating at 125 °C for 105 min according to ASTM D4426-01 method (reapproved 2006). Free formaldehyde content in the adhesives was determined via a hydroxylamine hydrochloride method in accordance with European Standard EN ISO 9397.

##### Thermogravimetric Analysis (TGA)

Thermal stability of the adhesives was tested via thermogravimetric analysis (TGA, Pyris 1 TGA, PerkinElemer, Shelton, CT, USA). Before the tests, all the samples were cured at 140 °C and ground into powder. For each TGA run, 10.0 mg of powder sample was loaded into a platinum pan and heated from 50 °C to 800 °C at 10 °C/min in N_2_ with a flow rate of 20 mL/min.

##### Adhesives for 3-Ply Plywood Lamination

The prepared adhesives were applied in bonding yellow birch veneers into 3-ply plywood for estimating bonding strength. Yellow birch veneers (11 × 11 × 1/16 inch^3^) were first conditioned at 20 °C and 65% RH for 7 days. The adhesives were mixed with wheat flour (10 wt% based on adhesive) as a filler. The mixture was then spread on the conditioned yellow birch veneers surface at the spread rate of 200 g/m^2^ for each glueline. The surface veneers were bonded perpendicularly with center veneer at 140 °C under 3.0 MPa for 4 min. Plywood specimens were cut for the mechanical test according to ASTM D 906-98. One-half of the specimens from each adhesive bonded plywood was conditioned at 20 °C and 65% RH for 7 days, followed by the mechanical test. While the other half was cooked in boiling water for 3 h, then cooled down in fume hood. All the 3-plywood specimens were tested at room temperature on shear stress of the glueline by tension loading with a benchtop universal testing machine (ADMET eXpert 7603 eP2 Universal Testing System) at a tension rate of 3 mm/min till failure. Honestly significant difference (Tukey’s HSD method) was applied for the statistical analysis among the mechanical testing results. In a given figure, values or bars annotated with the same letter mean insignificant difference at a 95% confidence level.

## 3. Results and Discussion

### 3.1. Basic Properties

As illustrated by Table 2, Alcell lignin-based adhesives show similar pH and non-volatile contents to NPF. While Alcell lignin-based adhesives exhibited higher free formaldehyde contents than NPF. Residual free formaldehyde content in lignin-based phenolic adhesives can be reduced by optimizing the formaldehyde/lignin ratio during lignin activation and the formaldehyde/phenol ratio during the adhesive synthesis. Relatively higher viscosity of lignin-based adhesives than that of NPF is observed and the addition of ML during adhesives synthesis at the second stage contributes to extremely high viscosity.

### 3.2. Thermal Stability

**Figure 2:**
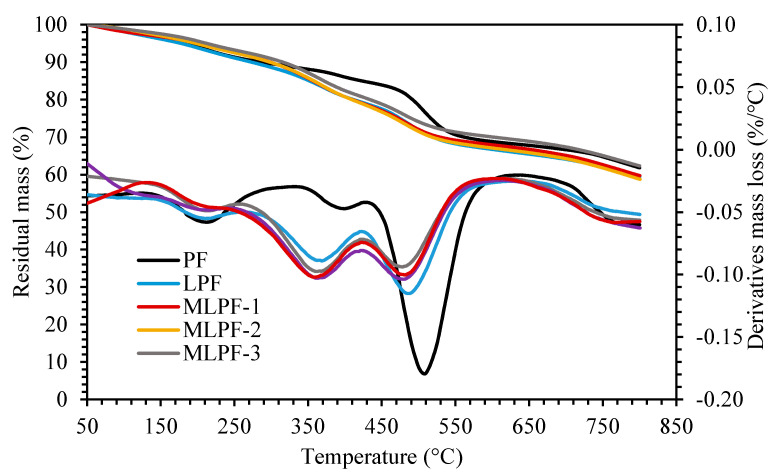
TGA and DTG profiles of the cured adhesives.

TGA and derivative thermogravimetric (DTG) profiles of the cured adhesives are as displayed by Figure 2. All the adhesives display typical three-stage degradation characteristics of phenol formaldehyde adhesive as we observed previously [18]. NPF shows three degradation peaks at 210 °C, 404 °C and 509 °C, respectively. The similar thermal degradation peaks were observed on the lignin based phenolic adhesives at approximately 210 °C, 360 °C and 490 °C, respectively. Compared with NPF, the associated degradation peaks shift to lower temperature levels, due to the less thermal stability of lignin based adhesives. Moreover, the solid residue of NPF and lignin based adhesives ranges in 58–62%, suggesting the similar fixed carbon content in all adhesives.

### 3.3. Bonding Strength of the Bonded 3-Ply Plywood

**Figure 3:**
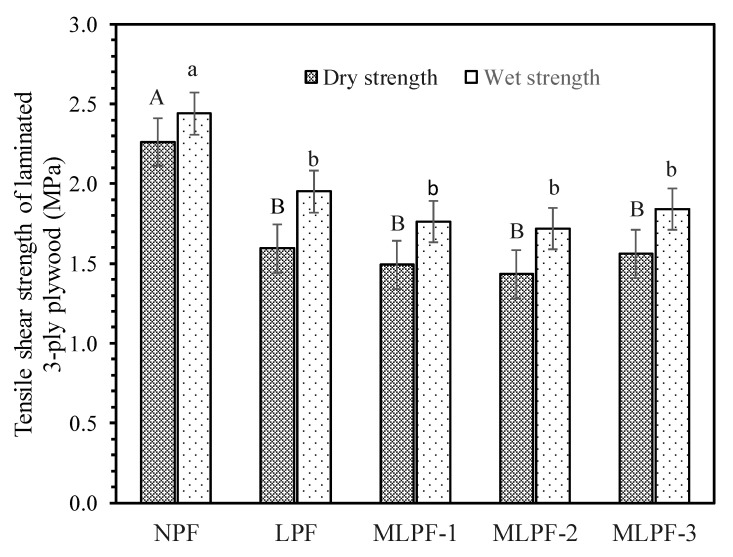
Tensile shear strength of 3-ply plywood bonded with the adhesives.

Bonding strength of 3-ply plywoods bonded by the adhesives is displayed in Figure 3. In contrast to the reported data, wet strength is higher than the corresponding dry strength for all the adhesives, even after 180 min of cooking of the plywood specimens in boiling water. This could be due to the further curing or cross-linking reaction of adhesives as well as the complete release of interior stress in the glueline during the cooking process. NPF adhesive can withstand up to 2.26 MPa and 2.44 MPa of tensile shear stress at dry and wet conditions, respectively. Lignin introduction into phenolic adhesives reduces the bonding strength when applied for plywoods. LPF displays a tensile shear strength of 1.59 MPa and 1.95 MPa at dry and wet conditions, respectively. For the associated MLPF-1, the bonding strength at dry and wet conditions is 1.49 MPa and 1.76 MPa, respectively. Lignin methyolation does not contribute to increased bonding strength compared with LPF. Addition of ML into adhesive at the second stage of adhesives synthesis results in further decreased bonding strength of 1.43 and 1.71 MPa at dry and wet conditions. While direct blending of ML and NPF for adhesive purpose contribute to comparable dry and wet bonding strength of 1.55 MPa and 1.84 MPa. Despite the above observations, lignin-based adhesives all meet the requirement for ASTM standard. Honestly significant difference analysis demonstrated that difference between bonding strength of NPF and any lignin based adhesive is significant. Meanwhile, regardless of methylolation or adding stage, difference among the lignin based adhesives is not significant as far as the bonding strength is concerned.

## 4. Conclusions

Alcell lignin was activated with sodium hydroxide and successfully integrated into phenolic adhesives at various stages. All lignin-based adhesives display both higher viscosity and free formaldehyde content than the neat phenol formaldehyde adhesive. When methylolated lignin was introduced during the second stage of adhesive synthesis, the resultant adhesive is much more viscous than any other adhesives. When exposed to heat at 50–800 °C, all the adhesives display typical three stage degradation characteristics, even though lignin-based adhesives show lower thermal stability than neat phenol formaldehyde adhesive. Bonding strength of lignin based adhesives all meet the requirement for ASTM standard, but bonding strength of any lignin based adhesive is significantly lower than that of neat phenol formaldehyde adhesive, regardless of dry or wet condition. While regardless of lignin methylolation or adding stage, difference among the lignin based adhesives is not significant with respect to bonding strength.

## Figures and Tables

**Table 1:** Elemental composition of Alcell lignin.

	Elemental Composition (wt%, d.b.^1^)				
	C	H	N	O ^2^	Ash ^3^
Organosolv lignin	71.60 (0.14)	6.30 (0.01)	0.17 (0.00)	21.90 (0.11)	2.68 (0.08)

^1^ On a dry basis. ^2^ Determined by the difference between 100% and total carbon/hydrogen/nitrogen/ash contents. ^3^ Determined by direct combustion at 575 °C.

**Table 2:** Basic properties of the adhesives.

	pH	Viscosity (50 °C, cP)	Non-Volatile Content (%)	Free Formaldehyde Content (%)
NPF	10.91	56.7 (3.7)	46.6 (0.2)	0.06 (0.002)
LPF	10.73	144.5 (4.9)	45.3 (0.5)	0.31 (0.019)
MLPF-1	10.74	131.3 (6.3)	44.7 (0.4)	0.41 (0.022)
MLPF-2	10.80	181.9 (10.9)	45.4 (0.3)	0.36 (0.032)
MLPL-3	11.17	131.3 (6.3)	44.5 (0.2)	0.43 (0.017)

## Data Availability

Not applicable.

## Sample Availability

Samples of the compounds are available from the authors.
